# Medical Students: A reflective journey over time

**DOI:** 10.15694/mep.2019.000105.2

**Published:** 2019-09-10

**Authors:** Saniya Sabzwari, Kulsoom Ghias, Anjiya Sulaiman

**Affiliations:** 1 The Aga Khan University; 2 The Aga Khan University

**Keywords:** Medical education, Medical students, Learning environment

## Abstract

This article was migrated. The article was marked as recommended.

Experiences in medical education have changed significantly in the last two decades. This evolution is apparent in the teaching/ learning environment, types of education resources and learners. In this article two students provide a reflective account of their individual journeys in medical education spanning across 20 years. While some things remain unchanged, significant differences emerge that stress upon the educators and institutions to stay mindful of these transitions in order to develop learning environments that nurture the growth of future physicians. Experiences in medical education have changed significantly in the last two decades. This evolution is apparent in the teaching/ learning environment, types of education resources and learners. In this article two students provide a reflective account of their individual journeys in medical education spanning across 20 years. While some things remain unchanged, significant differences emerge that stress upon the educators and institutions to stay mindful of these transitions in order to develop learning environments that nurture the growth of future physicians.

## Introduction

Higher education, in general, has shifted to a consumer model. A similar trend has been seen in medical education, attributed to increasing tuition fee and a sense of entitlement among students ( Eley and Stallman, 2014). Other changes in medical education are also well documented. Educators have highlighted differences in personality and attitudes between students of various generations ( Howe and Strauss, 2009; Borges *et al*., 2010). Learning and communication styles of students have also evolved with use of technology ( Bennett, Maton and Kervin, 2008). Furthermore, changes in the healthcare environment ( Garrick *et al*., 2019) have also impacted learning experiences of medical students ( Kilminster and Jolly, 2001). All of these changes require due consideration in developing and delivering medical curricula.

Do apparent differences widen the generational gap between educators and students, or are the recollections of the educators of today sheer nostalgia? ( Markman, 2011). Have student personalities and learning styles evolved enough to impact curriculum design? Are the goals and aspirations of medical students today drastically different?

A comparison of student experiences across two generations may help bridge the divide and inform an engaging curriculum to support student learning. Here we present the reflections of two students of a private medical college in Pakistan, the first from a graduate of the 1990s and the second from a student due to graduate in 2019.

## Reflective accounts

### The Generation X student

Sitting in seemingly colossal auditorium as initial introductions were made is the very first memory of my journey in medicine. The day seemed long and my eyes searched for familiar faces to stay (what I would later learn) my tachycardia.

In those days, courses in basic sciences were stand-alone subjects allowing in-depth study of these sciences related to organ systems. I recall anatomy sessions most vividly; the dissection hall became my favorite place to learn as the pungency of formaldehyde dissipated. The glaring nakedness of the body lying before me gave way to a quick sense of ownership of my cadaver whom our group cut through with curiosity; realizing how similar we all were from inside. A sense of camaraderie developed at these dissection tables where in detection of structures and organs we not only mastered anatomy, but also became acquainted with our own vulnerabilities. The excitement of discovery and the inevitability of end-of-life were lessons I learnt in that cold hall that formed the basis of my life as a physician-to-be.

Lab sessions offered another opportunity for hands on learning, at times however these seemed like frenzied experiments that had to be done right. Overall, the value of these lab sessions lay in their ability to teach the value of precision and organization. The volunteers for physiology experiments were often our male colleagues especially for experiments like gastric pH testing and electrophysiology of muscles. With current rigor in ethics of experimentation, changing societal norms, emphasis on gender equality such participant selection would be not be considered favorably.

Despite our significant immersion in experiential learning, lectures served as a key teaching strategy in our time. Even back then lectures catered less to the interest of the masses. These early morning talks brought out the disciplined and while the sleepy and the disheveled dragged their feet in to attend the sole source of interactive knowledge of its time.

There were always those who sat in front and diligently took notes and the chronic back-benchers who had mastered the art of dreaming with their eyes open, relying on their comrades’ lecture notes.

Back then, the curriculum was teacher-centered and passive learning was the norm. Note making was a reasonable alternate to thick textbooks allowing easier regurgitation of knowledge in exams. Despite a passive learning modality, some lectures allowed complete cognitive immersion. Some concepts learnt in such classes still seem to be just a thought away. Most teachers of that time were not only skillful communicators, but innate lovers of the art of education, imparting knowledge and instinctively engaging their audience. In their teacher led mode they still incorporated principles of student centeredness and managed to role model for us traits of generosity in instruction, inclusiveness of the average performer that some of us still strive to emulate in our role as educators today.

Two years flashed by bringing us to clinical years filled with excitement and occasional angst. Patient and inter-professional encounters made us realize that knowledge was no longer the sole criteria for success; humanistic traits and interpersonal skills were needed to survive and thrive.

Our student body was a menagerie of prodigies, the ordinary and the barely afloat students all neatly falling in a bell curve of academic performance. On the spectrum of social adeptness we had the brownnosers, the posers, the strategists and the vocally gifted all utilizing these unique strengths to gain favorable evaluations from faculty.

The initial weeks on the wards were a struggle to stay afloat on the sea of diagnoses, both mundane and exotic. Generally energetic and enthused, we learnt to ‘own’ our patients just like our consultants, most of whom helped refine our skills, but more importantly role modeled the traits of professionalism. Their interactions with patients not only earned our respect, but also shaped our career choices. Armed with an indispensable medical handbook as our instant go-to-guide, my classmates and I rounded for hours with and without our teams, immersing ourselves into learning. We were expected to know our patients and happily assumed responsibility of collecting blood samples and taking our patients for procedures. These tasks, though seemingly menial, helped us gain insight into the lives of our patients as we wheeled them down the long hospital corridors.

We communicated despite no emails, texting and social media, and always managed to assemble for hurriedly rescheduled sessions, at times waiting patiently for long hours in the hope of rounding with faculty.

Like others, I imbibed information from our final year students and residents who were mature, smart and confident **.** For an ingenuous student going through the initial year of clinical encounters, they were our messiahs, our most proximal instructors who took the time to explain pathophysiology and had our backs when we forgot lab results on our complicated patients. From them, I learnt what “See one, do one, teach one” meant.

In my present role of an educator, I can now appreciate that our zone of proximal development as described by Vygotsky was wide and deep, from nurses to seniors to peers and of course, the consultants. My learning was personal, interactive and immersive. We were fortunate to train in the days of the giants of medicine, the expert diagnosticians who with meticulous care teased apart symptoms and often reached accurate conclusions with very little help from tests and technology. Those were the golden years of medical education and hands on learning where the stress of patient volumes and revenue generation were not predominant determinants of care provision.

### The Generation Y student

I entered medical school with a seemingly endless supply of apprehension, nervousness, and excitement. The orientation week went by in a blur, but certain moments continue to stand out; a returned smile from a classmate who would eventually become a close friend, standing in a grand auditorium packed with nervous students and equally nervous parents, my final evenings spent with my family before they returned home. On that first day, we all milled around like lost puppies, tails wagging and enthusiastic smiles plastered on our faces. I found out in that first week that there were already class groups on various social media platforms, on which everyone had started communicating with some awkwardness. Over the years, these groups thrived and grew from stilted beginnings to bonding over jokes, tips and tricks about how to survive clerkships, and official announcements- all imperative for surviving medical school.

I considered myself a highly motivated individual. I had done well both in both the social and academic realms during my time in high school, and expected to carry on in more or less the same fashion at medical school. Seeing the poor results of my first examination was shocking. While coming to terms with the sudden dip in my academic performance, I began to curate social interactions that would lead to the formation of a successful support structure. There were so many platforms to juggle; the class Facebook group that everyone was joking on, constant surge of messages on WhatsApp groups, and real, face-to-face interactions sprinkled in between. Technology has become increasingly integrated the lives of my generation, to the point where there is no separating it from social or academic spheres.

The learning in the first two years are via discussion and debate, a problem-based learning system that brings students and faculty together to delve into topics in depth. I learnt to work hard, to cover for others when they didn’t manage to make it through all of the material, and be covered for in turn whenever I felt too lazy to finish my own work. I sat in varied groups some with students bickering and talking over one another and others that felt like old friends coming together. The interaction with faculty outside of these discussion sessions was not a significant part of my learning experience during my first two years. Anatomy labs were a tedious exercise of staring at PowerPoint slides and on rare occasion looking at cadaveric remains to try to comprehend human anatomy. With easy access to a variety of learning resources via the internet, non-interactive lecture sessions became an exercise in patience, with little student participation or interest. Pocket handbooks and notes provided bite-sized information and became our preferred source of text-based learning.

There was an unexpected level of disorientation entering clinical years, a dawning realization that this was the time to focus. I found to my delight that I loved clinical work. There is a sense of power, underscored with an intense humility, in taking ownership of a patient, learning about their lives and how their disease impacts their lives. Many of my days are spent trailing teams around wards and clinics, taking detailed histories and notes on interesting patients. Although I enjoy days on the floor, often my classmates and I find ourselves dragging our feet behind the large patient care teams, safe in the knowledge that no one is likely to pay attention to us falling behind. As a result, during my third year, many learning opportunities were lost. This led to a weak grasp of clinical knowledge forcing me to scramble to refresh in later years. Interacting with faculty who were eager to teach was a joy. Interacting with those for whom students were beneath their notice became an experience on how to deal with and please those who have more power than you. More often than not, the consultants fall somewhere in the middle of the spectrum, many consumed by their harried clinical schedules than teaching students.

We have our academic geniuses who always know the most obscure pathologies, others who are focus on early research opportunities and those involved in co-curricular activities, while trying to stay on track with their academics. Our learning styles are determined by our set of priorities, be it academics, research, extracurricular activities, or board examinations for residency.

Technology is ingrained in my life as a medical student. I gain most of my knowledge through the internet, videos, and easily downloadable e-books. Reliable applications on my cellphone allow me to access information efficiently in between patients. However, with the vast array of knowledge at my literal fingertips, I have to make a conscious effort to find reliable learning resources. The ever present threat with use of technology is always there: be it sharing of undependable knowledge or ethical issues like sharing pictures taken in the OR and clinics (with or without consent) on social media. Too often I, and others in my generation, ignore these concerns, and continue in blissful ignorance without considering the possible ramifications of our actions.

Throughout all these experiences, the rigor of the educational journey we undertake in medical school remains unchanged.

## Discussion

**Figure 1.**
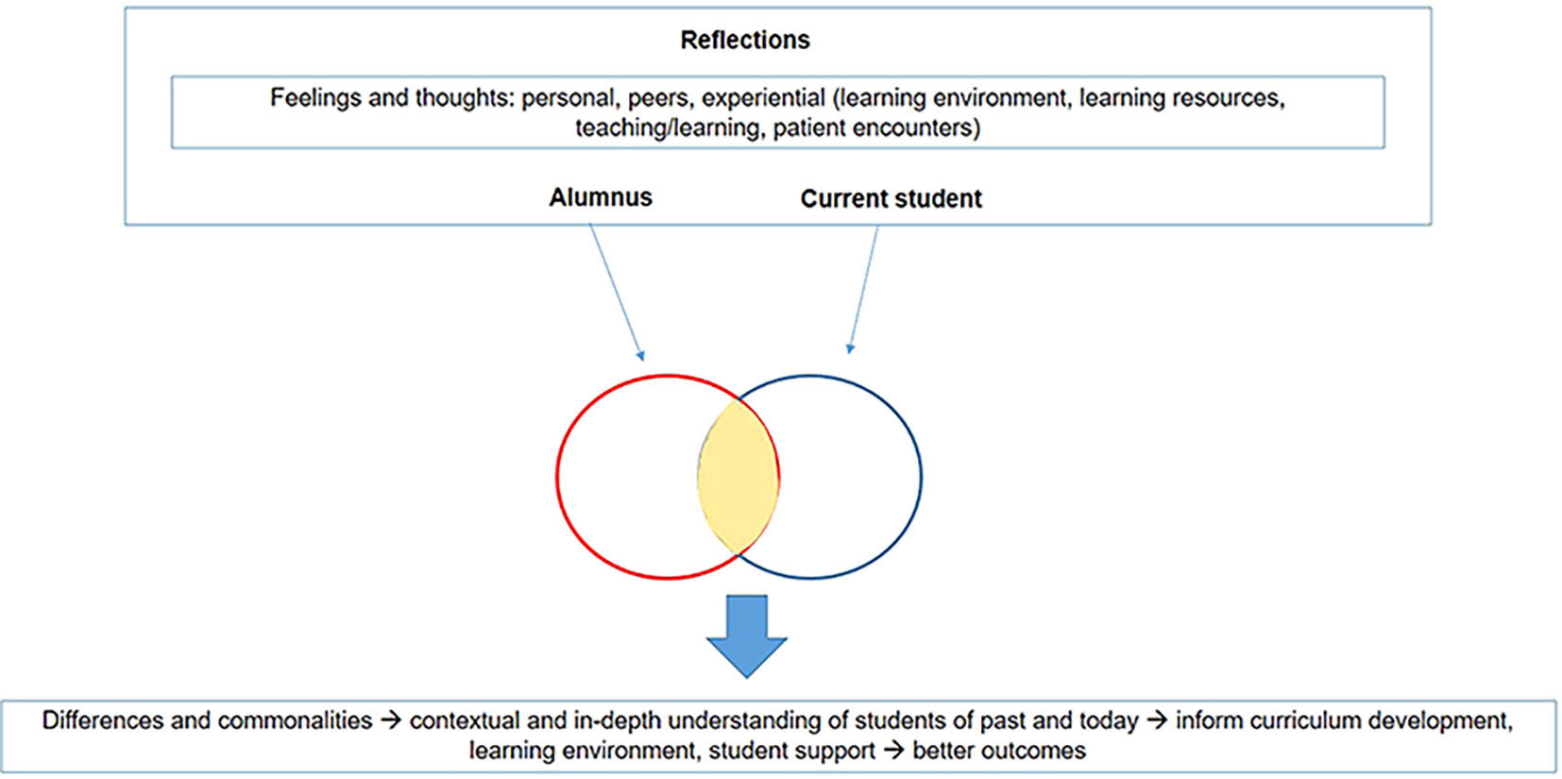
Framework of Analysis

The medical school experience has undoubtedly changed over the decades with the changing health system and hospital environment. The reflective accounts described here attempt to delineate generational differences and how changing medical school environments impact the learner, the learning and the curriculum. The framework of analysis used to identify themes from the reflections is depicted in Figure 1.

### Goals, aspirations and apprehensions

Though the differences in the student generations, curriculum and teaching learning styles are clearly evident, parallels can still be drawn. The same apprehension on entering medical school existed then and now, the melding of different personalities, the bonds formed through the journey still develop. The disappointments, some near misses, the joy of making a difference, the inspiration drawn from role models are experiences that continue to shape medical students across generations. The overall goal of medical students is still to succeed professionally, but the pathway to success is now influenced by changing post-graduate training opportunities ( USMLE, 2019) and evolving international standards for medical education ( WFME, 2019).

### Learning experiences

Unlike goals, aspirations and apprehensions, learning in medical education has changed significantly due to growing use and reliance on technology ( Vaona *et al*., 2018), the increasing workload at academic medical institutions ( West, Dyrbye and Shanafelt, 2018) and evolving medical education practice and philosophy. Ironically, when the curriculum was teacher-centered, the teachers were more student-centered. Faculty who have time and desire to teach are still considered “greats”, but these are fewer and far between because of competing academic and clinical expectations.

It appears that lectures were and are still are not favored by students ( Nevins *et al.,* 2016), but limited options three decades ago compelled students to attend lectures. In this day and age, there are multiple out-of-class learning options available on-demand to suit the learner’s schedule and preferences. The use of notes still exists, but textbook use has changed. Students now prefer using review guides and learning from “packets” of information rather than extensive text ( Scott, Morris and Marais, 2018). This move to using technology for learning has not been as actively embraced by faculty ( Kim, Kang and Kim, 2017) creating a disparity in expectations of learner and teacher. Moreover the over-reliance on technology appears to pose a valid threat to experiential learning, an essential component of medical education ( Delgaty, Fisher and Thomson, 2017).

On the clinical side, the student experience has become more peripheral and less immersive during patient care. Whereas students in the past contributed to even menial tasks related to patient care, the inadequate positive role modeling may be a factor in limiting student involvement during clinics and rounds. Lack of meaningful interaction of faculty with patients due to increased patient loads and competing demands set the stage for superficial student-patient contact.

### Connections and communication

The peer network continues to thrive and is a safety net for students. Although technology and social media provide opportunities to connect instantaneously, long-lasting bonds still take time to develop through sustained interpersonal interactions.

### Limitations

As a reflective paper, this manuscript shares the thoughts of only two students from different generations. Another limitation is that the reflections of the older student may have been influenced by her present day experience and role in medical education.

However, the two narratives do draw from observations and common experiences that were shared with their peers and are grounded in a framework of analysis and supported by literature for more objectivity.

### Way forward

The current generation of learners have different learning styles ( Twenge, 2009). Faculty members need to be more cognizant of these generational “learning” differences. This may be achieved through formal training on newer learning styles and techniques allowing faculty to better connect with students.

As key stakeholders, students need to be encouraged to use reflective practice to become learners who are responsible for their own learning. In today’s day and age, while students can curate their own knowledge, medicine cannot be learnt from the internet. Clinical teaching has been compromised and there is a need to strengthen communities of practice within clinical settings to allow for development of safe competent and professional physicians ( Kenny, Mann and MacLeod, 2003).

Clarification of faculty roles within institutions is needed in order to improve the balance between academic and clinical responsibilities. With the changing clinical milieu and increasing academic expectations from faculty, the need for such a balance becomes imperative to ensure that meaningful educational experiences continue.

## Conclusion

Through the reflective exercise documented here, we have identified generational differences that impact student didactic and experiential learning, as well as similarities which have persisted and can perhaps be used to bridge the gap between generations. How these differences shape future physicians and thereby impact patient care and health systems should be determined through systematic studies and analysis.

## Take Home Messages

• Medical education has undergone significant changes over time
• Teaching/learning methods have changed
• Learners and learning styles have changed
• Reliance on technology in medical education is increasing
• Academic medical institutions have to evolve to accomodate the changing milieu of health systems

## Notes On Contributors

SANIYA SABZWARI is an Associate Professor in the Department of Family Medicine at the Aga Khan University (AKU), Karachi. She has a Masters in Health Professions’ Education. She is currently the Co-chair of the Undergraduate Medical Education Curriculum Committee at AKU and has an interest in curriculum development and assessment, program evaluation, and student/faculty engagement. ORCID: https://orcid.org/0000-0002-1163-5277

KULSOOM GHIAS is an Associate Professor in the Department of Biological and Biomedical Sciences at the Aga Khan University (AKU), Karachi. She is currently the Co-chair of the Undergraduate Medical Education Curriculum Committee at AKU and has an interest in curriculum development and evaluation, and student engagement. ORCID: https://orcid.org/0000-0002-8709-7428

ANJIYA SULAIMAN is a final year Medical student at the Aga Khan University (AKU), Karachi. She has a keen interest in reflective and narrative writing and is also engaged in student support programs at AKU.
