# Supplementary material for: Medical Students: A reflective journey over time
Source: MedEdPublish (2016). 2019 Sep 10;8:105. Originally published 2019 May 17. [Version 2] doi: 10.15694/mep.2019.000105.2 (PMC10712461; doi:10.15694/mep.2019.000105.2)
Supplement: Peer review history

# Reviewer report: Reviewer response for version 2

**DOI:** 10.21956/mep.20248.r31409

**Published:** 2020-10-03

**Authors:** Shaoting Feng

**Affiliations:** 1 The First Affiliated Hospital of Sun Yat-sen University

This review has been migrated. The reviewer awarded 4 stars out of 5 Though the abstract is duplicated, it is still an interesting paper to read and reminding me of those days when I entered the medical school and started my journey on medical study as a millennium medical students.It is worth being recommended.

Reviewer Expertise:

NA

No decision status is available

# Reviewer report: Reviewer response for version 2

**DOI:** 10.21956/mep.20248.r31407

**Published:** 2019-11-26

**Authors:** P Ravi Shankar

**Affiliations:** 1 American International Medical University

This review has been migrated. The reviewer awarded 4 stars out of 5 I enjoyed reading this manuscript consisting of the reflection of two students of different generations. The manuscript has been beautifully written. Technology is having a major impact on the learning of medical students. The influence on other aspects of life is also notable. Instantaneous communication and a wealth of learning resources are available today. The influence of technology on medical education and practice will continue to grow. When I joined medical school we were totally dependent on the resources available in our medical school. In Anatomy we struggled to understand muscle actions and joint movements. Today there are excellent video resources available to illustrate the same. Another problem area was embryology for which also excellent animations are available. I agree with the authors that the traditional medical curriculum and the academic mindset have not kept up with the rapid advances in technology. There are a number of companies which are creating educational resources for medical school. Most of these are cloud-based and available with an institutional or an individual subscription. To a large extent I believe the internet has somewhat leveled the playing field allowing students everywhere to access first-rate learning resources. The article abstract is duplicated.

Reviewer Expertise:

NA

No decision status is available

# Reviewer report: Reviewer response for version 2

**DOI:** 10.21956/mep.20248.r31405

**Published:** 2019-10-18

**Authors:** Trevor Gibbs

**Affiliations:** 1 AMEE

This review has been migrated. The reviewer awarded 4 stars out of 5 An interesting paper to read and note- we need to hear from our students! It was just two students but I feel that this was a very useful piece of reflection, the only element missing was the "and so what now" one.It was a reflective piece written for good intentions and although there were a few very minor issues that might not have passed muster in some journals, I do think that this paper should be read by all those involved in curriculum planning

Reviewer Expertise:

NA

No decision status is available

# Reviewer report: Reviewer response for version 2

**DOI:** 10.21956/mep.20248.r31406

**Published:** 2019-09-12

**Authors:** Ryan Denkewicz

**Affiliations:** 1 Frank Netter School of Medicine at Quinnipiac University

This review has been migrated. The reviewer awarded 3 stars out of 5 The authors raise a number of interesting differences (and similarities) in the medical school learning environment between a Generation Xer and a Generation Yer. The paper could have benefitted from a deeper discussion about how the differences benefitted or disadvantaged them as students. Additionally, a survey of other Gen X and Gen Y medical graduates would have been helpful to provide more texture around the two different educational periods and how the historical changes have altered, if at all, educational outcomes. The paper left me reminiscent of my own medical school experience. It is a worthwhile read for those involved in the education of healthcare professionals.

Reviewer Expertise:

NA

No decision status is available

# Reviewer report: Reviewer response for version 2

**DOI:** 10.21956/mep.20248.r31408

**Published:** 2019-09-11

**Authors:** Ken Masters

**Affiliations:** 1 Sultan Qaboos University

This review has been migrated. The reviewer awarded 4 stars out of 5 I can see that the authors have addressed the issues raised by the reviewers of the first version. Unfortunately, the Abstract still appears duplicated, as it was in the first version of the paper. In addition, there are still areas where the discussion should be supported by the literature.Also, in the reflections from the elder student, the words “as described by Vygotsky” should have been placed in square brackets, so that the line reads: “I can now appreciate that our zone of proximal development [as described by Vygotsky] was wide and deep…” This is necessary because these words were not part of the original text, and were added into the text as a further explanation by the authors (because of reviewers’ comments).So, there are areas that could have been addressed more carefully, but it is an improved paper, and worth reading.

Reviewer Expertise:

NA

No decision status is available

# Reviewer report: Reviewer response for version 1

**DOI:** 10.21956/mep.19125.r27611

**Published:** 2019-05-30

**Authors:** Susan Van Schalkwyk

**Affiliations:** 1 Stellenbosch University

This review has been migrated. The reviewer awarded 2 stars out of 5 This article picks up on the debate about generational difference that has been part of the social fabric for millennia. The approach that the authors have adopted to offer different perspectives on the issue via the two reflective pieces is an interesting one. I do, however, feel that the piece as it stands could benefit from some revision, specifically in terms of drawing more directly on the relevant literature to strengthen the many claims that are made. I enjoyed reading the two narratives, but some critical reflection by the authors on the extent to which they can provide anything other than a prompt for further investigation, is needed. The categorization of current students as ‘digital natives’ and faculty staff as ‘digital dinosaurs’ or ‘immigrants’ is problematic and requires some critical consideration. See also the use of a 2008 reference to substantiate the claim about ‘students of today’. Ultimately, the insights offered in the discussion seem arbitrary and would have had more standing if relevant literature had been drawn into the text.

Reviewer Expertise:

NA

No decision status is available

# Reviewer report: Reviewer response for version 1

**DOI:** 10.21956/mep.19125.r27612

**Published:** 2019-05-25

**Authors:** Ken Masters

**Affiliations:** 1 Sultan Qaboos University

This review has been migrated. The reviewer awarded 2 stars out of 5 The paper draws on the experiences of two students at medical school, separated by 20 years, and discusses the implications in light of developments in medical education.A reflective paper where, essentially, n=2, is always hard to judge, and harder to rate highly. In order for the authors to ensure that their paper has some strength beyond musings, they have to ensure that the experiences of the two students are considered with some deep reflection, and with reference to the literature. In addition, in the areas where the authors write outside the students’ personal experiences, they need to make those sections very strong. In this paper, the students’ descriptions are well-written. The surrounding areas of text, however, need far more strengthening. In particular, here are some problems with the paper that need addressing:• The authors appear to have written the abstract twice. This needs to be corrected.• The introduction opens with two very controversial statements, neither of which are supported by cited literature. The authors will need to support these statements and/or modify them.• Similarly, in the Discussion, there are many generalisations made, and almost none of them are supported by any citations. (“Students now prefer using review guides and learning from "packets" of information rather than extensive text”, “Moreover the over-reliance on technology appears to pose a valid threat to experiential learning an essential component of medical education”, “the student experience has become more peripheral and less immersive during patient care.” These, and several more, either need to be modified or supported by literature, otherwise this discussion quickly devolves into a loosely-structured musing more suited to a personal blog than a personal opinion in an academic journal. Somewhat interesting, but not particularly valuable. • The term “Digital Natives”, while a useful general term, needs to be acknowledged as not entirely supported by the literature. In addition, if the authors are going to use the term, then it would be best to cite the originator of the term as the source (Prensky).• The first (older) student refers to “our zone of proximal development.” The fact that this student uses the term from Vygotsky so easily without even seemingly being aware of the fact that it is not normal parlance is a red flag for the authors. Not only is this student much older, but has far more knowledge and experience than most other students, and this knowledge and experience must surely indicate that the student is remembering their student days as filtered through these years of other experiences. As a result, one needs to question the accuracy of their reflection, given that self-reporting suffers from accuracy problems anyway; longer-term self-reporting even more so. It is not that the first student is lying; it is more that long-term recollections are not as accurate as short-term, and would undoubtedly be clouded by experience, and this has been ignored by the authors. It would have been worthwhile if the authors had picked this up in their Discussion, as they appear to be largely ignoring the impact of the passage of time on the accuracy of the recollections. So, this paper has the kernel of something useful, but the authors need to strengthen the literature context, and need to reflect upon (and take into account) the weakness of comparing older memories, filtered through experience, with those that are far more recent.

Reviewer Expertise:

NA

No decision status is available
